# Factors affecting hospital personnel’s disaster management performance

**DOI:** 10.1186/s12873-026-01597-6

**Published:** 2026-04-24

**Authors:** Burak Kaya, Sefa Mızrak

**Affiliations:** 1 Civil Defense and Firefighting Program, Bozkurt Vocational School, Kastamonu University, Kastamonu, Türkiye; 2 Emergency Aid and Disaster Management Department, Faculty of Health Sciences, Gümüşhane University, Gümüşhane, Türkiye

**Keywords:** Coordination, Disaster preparedness, Disaster management, Management performance

## Abstract

**Background:**

Hospital personnel with strong disaster management skills will work effectively and efficiently during disasters, ensuring better health services for staff, patients, and injured individuals.

**Purpose:**

This study aimed to investigate the factors affecting the hospital personnel disaster management performance (HPDMP) of Giresun Training and Research Hospital (Türkiye) according to the opinions of all hospital personnel.

**Method:**

In this study, survey data were collected from 797 hospital personnel between July and August 2023. HPDMP was employed as a dependent variable. Demographic characteristics, working conditions of personnel, disaster education, awareness of hospital disaster plans, and hospital disaster plan duty were used as independent variables. In addition, the arrangement of the hospital in relation to disaster risks, personnel self-efficacy, personnel coordination, coordination with other institutions, and disaster preparedness were also employed as independent variables.

**Results:**

The hospital’s disaster management performance was good. Personnel who received sufficient disaster education and had more knowledge about hospital disaster plans believed that HPDMP was greater. According to hospital personnel, disaster preparedness and coordination with other institutions were the factors that most strongly increased HPDMP.

**Conclusion:**

This study will provide insight for scientists, decision makers, and practitioners working in the field of hospital disaster planning and management.

**Supplementary Information:**

The online version contains supplementary material available at 10.1186/s12873-026-01597-6.

## Introduction

The structural and managerial resilience of hospitals in disaster-affected areas is crucial for providing effective services to affected people during and after a disaster [1]. The World Health Organization and the Pan American Health Organization [2] developed the Hospital Safety Index, which serves as a guide for health system managers, hospital administrators, personnel, and decision-makers, outlining the necessary preparations for hospitals to respond to and recover from emergencies and disasters. The index generally evaluates hazards affecting the hospital, structural and non-structural security measures, staff coordination capacity, the hospital disaster plan, information and communication management, human resources, logistics and financial status, maintenance and support services, and security measures. Hospitals that develop disaster preparedness plans, regularly revise them, and increase awareness of these plans among all personnel through training and drills are more resilient to disasters [3].

Hospitals have complex structures and are exposed to both internal and external crises in terms of environmental, physical, and social aspects [4]. Disasters disrupt the functioning of society and settlements, causing an increase in demand for healthcare institutions and increasing the need for healthcare personnel and organizations [5]. Information and communication technology failures, internal and external fires, power outages, hazardous materials, structural damages, and violent incidents in hospitals cause crises and service disruptions [6]. For example, after the 2023 Kahramanmaraş earthquake in Türkiye, many hospitals were destroyed or damaged, causing more loss of life in the earthquake area [7]. Since disasters destroy hospitals, disaster victims are transferred to hospitals in other regions [8]. This situation causes congestion in evacuated hospitals, increasing the need for manpower and supplies [9, 10]. Therefore, the adequacy of the existing health system is critical for reducing the loss of life as a result of mass events and disasters [11].

The prevalence of disasters in the world has increased the importance of disaster management activities in hospitals [5]. Hospitals must be durable and functional against disasters and emergencies [12]. Necessary preparedness should be implemented in hospitals and other healthcare institutions to prevent damage that may occur in the event of disasters [13–15]. To be more effectively prepared for disasters, hospitals should have disaster and emergency preparedness plans [16]. These plans should cover all risks and hazards that may affect the hospital [14, 17]. A lack of medical equipment and supplies in hospitals can cause greater problems during disasters [18]. Disaster education, practices, planning, and early warning systems in hospitals will ensure the continuation of health services during disasters and will accelerate the hospital’s rapid return to normal operation [19]. Evaluating a hospital’s performance, especially after crises, will allow the creation of more reliable social and technical systems and well-adapted emergency preparedness [20]. Assessing the strengths and weaknesses of hospitals and developing improvement programs will enable them to manage disaster risks more effectively [21].

Public policies produce strategies to increase the capacity of hospitals to respond effectively and without interruption in crises and disasters. For example, the Organization for Economic Co-operation and Development (OECD) supports countries so that people can access human-centered, high-performance, resilient, and technology-based health systems [22]. The Sendai Framework for Disaster Risk Reduction 2015–2030 states that hospitals are among the critical infrastructures that need to be evaluated and strengthened in terms of economic, social, structural, technological, and environmental aspects to reduce disaster risks for resilience and to become better in the post-disaster process [23]. All hospital employees in Türkiye must be trained on basic disaster awareness, fire, reduction of non-structural hazards, chemical, biological, radioactive, and nuclear incidents, radiation protection, and disaster and emergency triage [24]. Hospitals in Türkiye are required to take disaster mitigation measures and prepare for disasters, and to carry out hospital disaster and emergency plans and drills to continue their services without any assistance from outside the hospital for the first seventy-two hours after a disaster [25].

This study aims to investigate the disaster management performance of hospital personnel (HPDMP) working in Giresun Training and Research Hospital in Türkiye. HPDMP meant that personnel provide effective and efficient treatment and care to the injured people in a disaster situation, in harmony with other personnel, at the ideal time. This study will investigate how the socio-demographic characteristics of the personnel, their working conditions in the hospital, their disaster education capacity, their awareness of the hospital disaster plan, and their duties in the plan are correlated with HPDMP. Additionally, personnel perceptions regarding the status of nonstructural risks in the hospital, personnel self-efficacy and coordination, coordination with other institutions, and hospital disaster preparedness will be determined, and the effects of these perceptions on HPDMP will be examined. The results of this study are important for the development of personnel skills, hospital disaster preparedness, coordination, and cooperation policies that will ensure the effective use of hospitals during disasters. Furthermore, the results will provide insights to local and national disaster managers, provincial health directorates, hospital administrators, hospital disaster plan developers, and other disaster management stakeholders on how to make hospitals safer against disasters.

## Method

### Research design

This study employed a correlational design, utilizing quantitative research methods. Correlational research reveals whether there is a relationship between variables, the amount of the relationship, and whether the relationship is positive or negative [26].

### Participants

This study was conducted at the Training and Research Hospital in Giresun, Türkiye. This hospital is the largest health institution in the province, with a capacity of 450 beds and 1678 personnel. The survey data were collected from all personnel working in the hospital. The first author of this study, who was responsible for the preparation and implementation of hospital disaster plans in this hospital, collected data through face-to-face interviews with each staff member. During the data collection process, the author explained to the participants the purpose of the study and the items in the survey instrument. Many personnel did not want to participate in the study. Some personnel were unable to participate in the study because they were on leave or temporarily working at another healthcare institution. Finally, 797 (47.4%) of the hospital staff were included in this study, and 881 (52.6%) staff were excluded.

### Survey instrument

The survey in this study consisted of three parts (Appendix 1). The first part determined the gender, age, education level, and marital status of hospital personnel. The second part determined the work experience, occupation, floor worked in the hospital, work schedule, disaster education qualification, hospital disaster plan awareness, and duty in the hospital disaster plan. In the third section, there were six items with a 5-point Likert scale (1 = strongly disagree, 5 = strongly agree). These items are as follows;

Nonstructural risks: Items within the hospital are arranged appropriately against disaster risks (for example, cabinets are fixed, devices are fixed).

Self-efficacy: I know what to do in case of a disaster in the hospital.

Personnel coordination: In case of a disaster, we can work in coordination with the personnel in my unit.

Coordination with other institutions: In case of disaster, our hospital staff can work in coordination with other institution personnel (Disaster and Emergency Management Directorate, Gendarmerie, Police, Fire Brigade, etc.).

Disaster preparedness: I think that in case of a disaster, the materials in my unit will be sufficient for at least three days.

Hospital personnel’s disaster management performance: In case of a disaster, your hospital personnel’s disaster management performance will be successful. (Imagine a major disaster in Giresun. Many injured people will come to your hospital. In such a case, how effectively and efficiently can the hospital personnel work with the least amount of problems to provide service to the injured? ).

The variables affecting the performance of hospital personnel in disaster management were formulated based on studies on hospital disaster management [27], resilience [28], preparedness [16, 29], safety [30], and recovery [31, 32]. Many studies found that the individual characteristics and working conditions of the personnel affect the service provided during a disaster [32]. The items placed appropriately to mitigate disaster risks in the hospital will not harm people or the environment during a disaster [12]. It is predicted that personnel who receive disaster training will work more effectively during disasters [23–25]. The coordinated and collaborative work of personnel from the same or different institutions enhances the speed and efficiency of disaster response [29, 33]. Sufficient medical supplies in the hospital after disasters are necessary for the continuity and quality of service in the hospital [16, 18]. Therefore, disaster response success will be high in hospitals that are prepared for disaster risks, have knowledgeable personnel, and have high team harmony.

By measuring these variables with a single item, it was hoped that the participants would evaluate the hospital as a whole comprehensively and easily. As the score obtained from these six items increases, the specified variables increase positively. After the survey was created, five scientists working in the field of disaster management examined the form scientifically and offered some suggestions. To check the clarity of the items, the survey was applied to 10 personnel working in different positions in the hospital where the study was conducted. The appearance and items of the survey were arranged according to the suggestions of both scientists and hospital personnel.

### Data analysis

The data were analyzed with descriptive statistics and ordinal logistic regression (OLR) using the SPSS program. HPDMP was used as the dependent variable. Since the dependent variable is progressively increasing and the independent variables are categorical and progressively increasing, OLR was employed. The suitability of the data for OLR was determined by assessing the variance inflation factor and tolerance values ​​to determine whether there was a multicollinearity problem. Since the variance inflation factor values ​​were below 4 [34] and the tolerance values ​​were above 0.2 [35], the data in this study were suitable for OLR. Three OLR analyses revealed how much and how the dependent and independent variables were correlated. In the first analysis, the sociodemographic characteristics of the personnel were used as independent variables. In the second analysis, the personnel’s working conditions in the hospital, disaster education, hospital disaster plan awareness, and their role in the hospital disaster plan were used as independent variables. In the third OLR analysis, nonstructural risks, self-efficacy, personnel coordination, coordination with institutions, and disaster preparedness were used as independent variables. The results of OLR analyses were interpreted according to odds ratios (ORs), 95% confidence intervals (95% CIs), and Nagelkerke R-squared values. An OR less than 1 indicates a negative correlation between the dependent variable and the independent variable, and an OR greater than 1 indicates a positive correlation between the dependent variable and the independent variable. Values of 0.001, 0.01, and 0.05 were considered to indicate statistical significance.

## Results

Table 1 shows the demographic information of the hospital personnel who participated in this study. A total of 797 hospital personnel working at Giresun Training and Research Hospital participated in the study. When the demographic distribution was evaluated in general, women and undergraduate graduates participated more in the study.

**Table 1:** Demographic characteristics of the hospital personnel participating in the study

Variables	Groups	Frequency	Percent (%)
Gender	Male	273	34.3
	Female	524	65.7
Age	20–25	180	22.6
	26–30	288	36.1
	31–35	107	13.4
	36 and over	222	27.9
Marital status	Married	410	51.4
	Single	387	48.6
Education	Secondary education or below	33	4.1
	High school	113	14.2
	Preundergraduate	206	25.8
	Undergraduate	395	49.6
	Master’s degree	36	4.5
	Doctorate	14	1.8

Table 2 shows the participants’ working conditions in the hospital and their status in receiving disaster education, awareness of the hospital disaster plan, and their role in the hospital disaster plan. The majority of the participants worked on the first two floors and 8 h a day, and nurses participated in the study the most.

**Table 2:** Distribution of hospital personnel participating in the study according to their work experience, occupation, floor they work in, working schedule, disaster education status, hospital disaster plan knowledge, and hospital disaster plan duty

Variables	Groups	Frequency	Percent (%)
Work experience	1–3 years	376	47.2
	4–10 years	217	27.2
	11 years or above	204	25.6
Occupation	Medical doctor	53	6.6
	Nurse	283	35.5
	Cleaning staff	134	16.8
	Secretary	107	13.4
	Technician	104	13.0
	Other health graduates	28	3.5
	Officer	51	6.4
	Security guard	37	4.6
Working floor	Basement floor	75	9.4
	Ground floor	228	28.6
	1st floor	198	24.8
	2nd floor	189	23.7
	3rd floor	24	3.0
	4th floor	33	4.1
	5th floor	21	2.6
	6th floor	29	3.6
Working schedule	8 h	523	65.6
	12 h	71	8.9
	24 h	203	25.5
Disaster education	No	151	18.9
	I received insufficient disaster education	161	20.2
	I received intermediate-level disaster education	348	43.7
	I received sufficient disaster education	137	17.2
Hospital disaster plan awareness	I have no knowledge	150	18.8
	I have intermediate knowledge	558	70.0
	I have a lot of knowledge	89	11.2
Role of staff in the hospital disaster plan	I have no duty	633	79.4
	Response team	30	3.8
	Rescue team	26	3.3
	Incident management team	19	2.4
	CBRN team	21	2.6
	I do not know if I have a duty or not	68	8.5

Table 3 shows the means and standard deviations of the nonstructural risks, self-efficacy, personnel coordination, coordination with other institutions, disaster preparedness, and HPDMP variables. The means of all variables were above the medium level. The mean of the coordination with other institutions variable was the highest, and the mean of the disaster preparedness variable was the lowest.

**Table 3:** Descriptive characteristics of nonstructural risks, self-efficacy, personnel coordination, coordination with other institutions, disaster preparedness, and HPDMP variables

Variables	Mean	SD
Nonstructural risks	3.365	1.268
Self-efficacy	3.518	1.009
Personnel coordination	3.690	1.025
Coordination with other institutions	3.880	0.952
Disaster preparedness	3.346	1.184
HPDMP	3.488	1.046

SD = Standard deviation

**Fig. 1:**
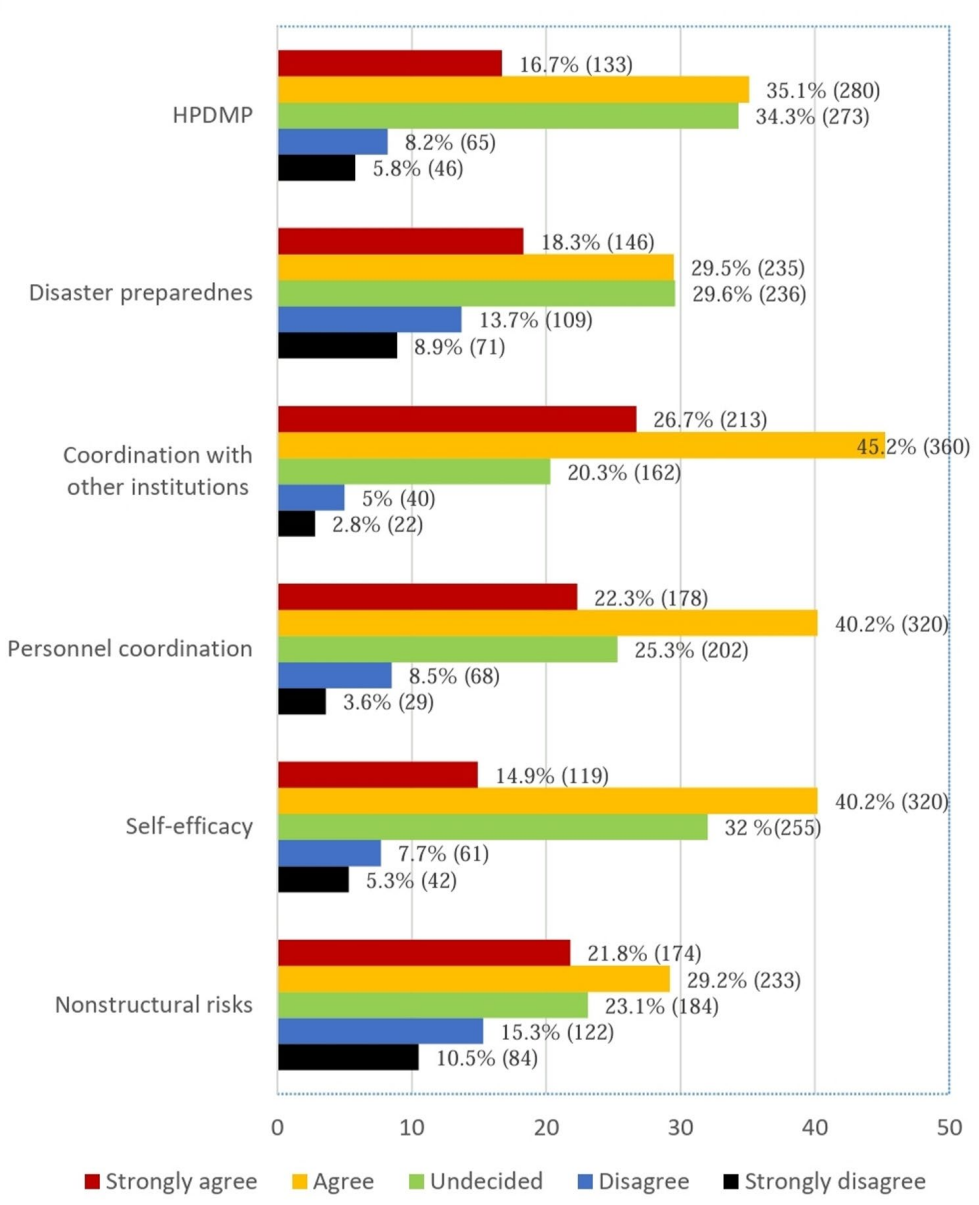
Response distribution of nonstructural hazards, self-efficacy, personnel coordination, coordination with other institutions, disaster preparedness, and HPDMP variables

Figure 1 shows the response distributions of the variables of nonstructural risks, self-efficacy, personnel coordination, coordination with other institutions, disaster preparedness, and HPDMP in percentages and numbers. A total of 21.8% (174) of the participants believed that the items in the hospital were not properly arranged to mitigate disaster risks. 5% (42) of the participants stated that they had no knowledge of what to do in case of a disaster in the hospital, and 14.9% (119) stated that they knew exactly what to do in case of a disaster. A total of 26.7% (213) of the participants thought that they could work in coordination with the personnel in the unit in case of a disaster. A total of 29.6% (236) of participants were undecided about the hospital’s disaster preparedness. While 16.7% (133) of the participants thought that the disaster management performance of the hospital was completely successful, 5.8% (46) thought that the disaster management performance of the hospital was not sufficient.

Table 4 shows the correlations between the demographic variables and HPDMP. Gender, marital status, and education were not significantly correlated with HPDMP. Among the age groups, only the 26–30 age group had a statistically significant correlation with HPDMP. The disaster management performance of the hospital perceived by the participants between 26 and 30 years of age was lower than HPDMP stated by the participants aged 36 and over (OR = 0.565, 95% CI = 0.398–0.804). According to the Nagelkerke R-squared value, demographic variables explained 7% of the variation in HPDMP.

**Table 4:** OLR results of the correlation between demographic variables and HPDMP

Independent Variables	Groups	ORs	95% CIs	
			Lower	Upper
Gender	Male	1.127	0.854	1.487
Age	Female^a^			
	20–25	0.885	0.576	1.360
	26–30	0.565***	0.398	0.804
	31–35	1.081	0.707	1.653
	36 and over^a^			
Marital status	Married	0.970	0.718	1.310
	Single^a^			
Education	Secondary education or below	2.702	0.858	8.507
	High school	2.291	0.829	6.333
	Preundergraduate	2.243	0.820	6.138
	Undergraduate	1.091	0.408	2.920
	Master’s degree	1.048	0.337	3.258
	Doctorate^a^			
**Pseudo R-Square**				
Nagelkerke		0.073		

a **=** reference category, ***p < 0.001, **p < 0.01, *p < 0.05

Table 5 shows the correlations between working conditions in the hospital, receiving disaster education, hospital disaster plan awareness, hospital disaster plan duties, and HPDMP. Work experience, occupation, floor worked, and duty in the hospital disaster plan are not significantly correlated with HPDMP. Hospital personnel working 8 h thought that HPDMP was greater (OR = 1.521, 95% CI = 1.050–2.203). Participants who received no disaster education (OR = 0.476, 95% CI = 0.287–0.789) and those who received insufficient disaster education (OR = 0.399, 95% CI = 0.247–0.645) stated that HPDMP was lower. HPDMP was lower for personnel who had no knowledge of hospital disaster plans (OR = 0.122, 95% CI = 0.066–0.227) and those who had moderate knowledge of hospital disaster plans (OR = 0.253, 95% CI = 0.152–0.421). According to the Nagelkerke R-squared value, the working conditions of the hospital staff, their status of receiving disaster education, and their duties in the hospital disaster plan explained 23.9% of the variation in HPDMP.

**Table 5:** OLR results of the correlation between work experience, occupation, floor worked, working schedule, disaster education, hospital disaster plan knowledge, hospital disaster plan duty, and HPDMP

Independent Variables	Groups	ORs	95% CIs	
			Lower	Upper
Work experience	1–3 years	1.075	0.758	1.524
	4–10 years	0.855	0.588	1.242
	11 years or above^a^			
Occupation	Medical doctor	0.880	0.367	2.110
	Nurse	0.631	0.296	1.343
	Cleaning staff	1.165	0.532	2.552
	Secretary	1.674	0.770	3.640
	Technician	0.954	0.434	2.099
	Other health graduates	1.122	0.401	3.140
	Officer	1.700	0.691	4.181
	Security guard^a^			
Working floor	Basement floor	0.608	0.257	1.438
	Ground floor	1.335	0.627	2.844
	1st floor	1.243	0.584	2.645
	2nd floor	0.995	0.468	2.114
	3rd floor	1.074	0.386	2.988
	4th floor	0.789	0.308	2.020
	5th floor	0.933	0.325	2.675
	6th floor^a^			
Working schedule	8 h	1.521*	1.050	2.203
	12 h	1.573	0.891	2.777
	24 hours^a^			
Disaster education	No	0.476**	0.287	0.789
	I received insufficient disaster education	0.399***	0.247	0.645
	I received intermediate level disaster education	0.788	0.521	1.193
	I received sufficient disaster education^a^			
Hospital disaster plan awareness	I have no knowledge	0.122***	0.066	0.227
	I have intermediate knowledge	0.253***	0.152	0.421
	I have a lot of knowledge^a^			
Role of staff in the hospital disaster plan	I have no duty	0.875	0.542	1.414
	Response team	1.312	0.557	3.091
	Rescue team	0.865	0.362	2.067
	Incident management team	1.055	0.388	2.869
	CBRN team	1.219	0.477	3.117
	I do not know if I have a duty or not^a^			
**Pseudo R-Square**				
Nagelkerke	0.239			

a **=** reference category, ***p < 0.001, **p < 0.01, *p < 0.05

Table 6 shows that there was a statistically significant and positive correlation between nonstructural risks, self-efficacy, personnel coordination, coordination with other institutions, and disaster preparedness and HPDMP. Disaster preparedness was the independent variable that increased HPDMP the most, while personnel coordination was the independent variable that increased hospital disaster management the least. Nonstructural risks, self-efficacy, personnel coordination, coordination with other institutions, and disaster preparedness explained 64% of the variation in HPDMP.

**Table 6:** OLR results of the correlation between nonstructural risks, self-efficacy, personnel coordination, coordination with other institutions, disaster preparedness and HPDMP

Independent Variables	ORs	95% CIs	
		Lower	Upper
Nonstructural risks	1.478***	1.300	1.680
Self-efficacy	1.596***	1.336	1.908
Personnel coordination	1.397***	1.141	1.710
Coordination with other institutions	2.267***	1.825	2.815
Disaster preparedness:	2.807***	2.404	3.277
**Pseudo R-Square**			
Nagelkerke		0.640	

a **=** reference category, ***p < 0.001, **p < 0.01, *p < 0.05

## Discussion

This study aimed to assess the disaster management performance of hospital personnel at Giresun Training and Research Hospital in Türkiye, as well as the factors influencing it. All hospital floors, departments, and personnel were included in this study; therefore, the results are valid for the hospital as a whole. The hospital’s and personnel’s capacity to combat disasters was presented descriptively, and the effect of these capacities on HPDMP was examined. This study indicated that among the socio-demographic characteristics of participants, only age was correlated with HPDMP. Personnel working eight hours a day at the hospital reported that the disaster management performance of hospital personnel would be high in the event of a disaster. Therefore, training and drills can be organized according to the working time of hospital personnel.

Personnel with high levels of disaster education and awareness of the hospital’s disaster plan believed that HPDMP would be more effective. Sarık and Cengiz [36] reported that personnel who received disaster education participated more in hospital disaster plan drills. Avcı et al. [37] reported that very few participants participated in disaster drills, and many were unaware of the existence of hospital disaster plans. Şen and Ersoy [38] revealed that some individuals responsible for hospital disaster management were unaware of their term of office. Paganini et al. [39] reported that many emergency department workers were unaware of the person responsible for the hospital disaster plan. Sa’d and Malak [40] found that the disaster management training program increased the knowledge, skills, and personal preparedness levels of emergency workers. Healthcare professionals highlighted that comprehensive, detailed, risk-reduction-focused, participatory, continuously improved, awareness-raising, and easy-to-implement hospital disaster plans increase the resilience of hospital healthcare systems [41]. On the other hand, many participants in this study stated that they had not received adequate disaster training and were unaware of the hospital’s disaster plan. These personnel may make incorrect and inadequate responses in a disaster situation or may work incompatibly with other personnel. Therefore, hospital administrators should ensure that all personnel receive the necessary disaster training and are completely aware of the hospital’s disaster plan.

The variables nonstructural risks, self-efficacy, personnel coordination, coordination with other institutions, and disaster preparedness were all positively and significantly correlated with HPDMP. Perrone et al. [42] observed that even if the building did not suffer severe damage during the earthquake, partition walls, ceiling strength, non-structural vaults, chimneys, and storage shelving were damaged. The February 6, 2023 Kahramanmaraş earthquakes caused non-structural damage inside or around many hospitals, rendering many areas unusable [43]. Failure to account for non-structural risks and take necessary precautions in a hospital could prevent the use of materials and equipment in a disaster situation and make evacuation more difficult.

The results of a study conducted in twelve hospitals showed that more than half of the participants could not take adequate precautions in case of a disaster and had insufficient knowledge and skills to implement evacuation procedures [44]. Janizadeh et al. [5] reported that many personnel with sufficient knowledge about disasters had poorer practical skills. Gerdan et al. [45] found that in the earthquake-affected region, hospital personnel’s crisis management skills and individual experiences influenced the sustainability and quality of healthcare services. Aslan [46] stated that the technical, operational, leadership, and behavioral skills of personnel during a disaster affected the response process and coordination with other teams. Hospital administrators should also consider personnel who perceive themselves as having low self-efficacy in this study. This is because personnel with low self-efficacy may not be able to provide adequate service and coordinate with other personnel. Since self-efficacy positively affects the performance of hospital personnel in disaster situations, training and drills tailored to different types of disasters should be organized to enhance their self-efficacy.

Health care personnel who responded to three terrorist incidents emphasized that strong coordination and communication among teams greatly increased the effectiveness of the responses [33]. Seyghalani Talab et al. [47] reported that support management, preparedness, post-crisis recovery, responsiveness, and adaptability during a disaster have a positive and significant impact on the organizational resilience of hospitals. De La Garza and Lot [20] revealed that effective crisis management, organization, information flow, mass mobilization, effective management of human resources, adequate preparation, and information in hospitals during the COVID-19 pandemic increased institutional resilience. Nurses in Türkiye who came to work in the earthquake-affected region from various institutions and regions reported that they encountered significant issues with management and coordination [48]. Samei et al. [17] stated that coordination, control, and command were the main factors affecting hospital preparedness for disasters. The garden, ground floor, and basement of the hospital damaged by flood water were cleaned with the timely intervention of municipal teams [19]. During the Kahramanmaraş earthquakes, civilian volunteers supported patients and healthcare personnel in hospitals [49]. A study conducted in thirteen hospitals in India reported that emergency response structures within hospitals and information sharing among external partners would enhance regional health disaster resilience [50]. To ensure the effective and efficient use of resources in the hospital, collaboration with other institutions should be increased. In particular, qualitative research should be used to investigate the opinions of hospital personnel who report low coordination levels regarding coordination problems, and solutions should be developed.

Disaster preparedness was the variable that most improved hospitals’ disaster management in this study, despite having the lowest mean. Health professionals in Yemen emphasized supply shortages as the main factor that can cause crises in hospitals [18]. After the Kahramanmaraş earthquake, many hospitals ran out of supplies, resulting in inadequate treatment for earthquake victims [51], and the medical supplies brought by the national health teams were depleted in a short time due to intense demand [7]. A study investigating the disaster and emergency preparedness of twenty-four hospitals in Lebanon revealed that more than half of the hospitals did not have disaster medical carts or disaster supplies [16]. Furthermore, in this study, many personnel stated that the hospital’s disaster preparedness was either nonexistent or inadequate. Therefore, deficiencies in disaster preparedness should be addressed by interviewing each unit, floor, and personnel in the hospital. Otherwise, effective services may not be provided in some parts of the hospital due to insufficient disaster preparedness.

## Limitations

This study was conducted in only one hospital, and less than half of the personnel participated in the study. Since each hospital has different physical, staffing, and environmental characteristics, the results are limited to the hospital studied. The single-item measurement method using 5-point Likert-type items enabled hospital personnel to express their thoughts more quickly and easily with a greater number of variables. However, they (nonstructural risks, self-efficacy, personnel coordination, coordination with other institutions, disaster preparedness, and HPDMP) are valid for the entire hospital, and therefore, they may differ according to units, floors, and occupations. These variables are multidimensional; however, they were superficially identified in this study. For example, coordination with other institutions was generally determined; however, there are many institutions with which the hospital must cooperate and work in coordination in case of disasters. Furthermore, there are many types of disasters that can affect hospitals, and this study focused on disasters in general. Therefore, a more comprehensive and detailed investigation of hospitals’ resilience, personnel capacity, equipment adequacy, and disaster management skills for each specific type of disaster will yield more thorough and detailed results. The study does not include how risks arising from the hospital’s structural features and environment may affect HPDMP. On the other hand, the hospital’s disaster management performance was evaluated solely based on the opinions of its personnel. Personal biases, prejudices, and knowledge of hospital disaster management may have influenced the results. To obtain more reliable results, hospital disaster managers should evaluate the hospital’s disaster management capacity through field and tabletop exercises. Through drills and disaster scenarios, the number of injured people who may arrive at the hospital in a disaster can be estimated, and the adequacy of personnel and materials can be tested.

## Conclusions

This study revealed that gender, marital status, and education were not significantly correlated with HPDMP. Work experience, occupation, floor worked, and involvement in the hospital disaster plan were also not significantly correlated with HPDMP. Personnel who work eight hours a day at the hospital reported that HPDMP was better. Personnel who reported receiving adequate disaster education and who believed they had a great deal of knowledge about hospital disaster plans also reported that HPDMP was better. Arranging the hospital’s belongings according to disaster risks, the personnel’s high disaster response capacity, the personnel’s ability to work in coordination with other institution personnel, and the hospital’s high disaster preparedness increased HPDMP.

All hospital personnel should receive adequate theoretical and practical training to prepare for crises that may be encountered inside and outside the hospital. To ensure the successful implementation of the hospital disaster plan during crises, personnel awareness of the plan should be increased. Hospital management should organize joint exercises periodically to strengthen cooperation and harmony among personnel and external stakeholders. Hospital disaster managers and scientists should work together to update hospital disaster plans and preparedness based on past disaster experiences.

## Supplementary Information

Below is the link to the electronic supplementary material.

Supplementary Material 1

## Data Availability

The data are available from the authors on reasonable request.

## Abbreviations

HPDMP: Hospital Personnel’s Disaster Management Performance
OR: Odds Ratio
CI: Confidence Intervals
SPSS: Statistical Package for the Social Sciences
OLR: Ordinal Logistics Regression

## References

[1] Fallah-Aliabadi S, Ostadtaghizadeh A, Ardalan A, Fatemi F, Khazai B, Mirjalili MR. Towards developing a model for the evaluation of hospital disaster resilience: A systematic review. BMC Health Serv Res. 2020;20:1–11. 10.1186/s12913-020-4915-2. 10.1186/s12913-020-4915-2 PMC6988294 31996213

[2] World Health Organization and Pan American Health. Hospital safety index: Guide for evaluators – 2nd ed. World Health Organ, (2015). https://iris.who.int/bitstream/handle/10665/258966/9789241548984-eng.pdf?sequence=1

[3] Mohtady H, Desha C, Ranse J, Roiko A. Planning and assessment approaches towards disaster resilient hospitals: A systematic literature review. Int J Disaster Risk Reduct. 2021;61:102319. 10.1016/j.ijdrr.2021.102319.

[4] Abbasabadi-Arab M, Khankeh HR, Mosadeghrad AM, Biglarian A. Comprehensive disaster risk management standards for hospitals, Heal. Emergencies Disasters Q. 2023;8:95–106. 10.32598/hdq.8.2.208.1

[5] Janizadeh R, Omidvari F, Motlagh Z, Jahangiri M. Knowledge, attitude, and practice of hospital staff. Heal Emergencies Disasters Q. 2023;8:185–92. 10.32598/hdq.8.3.482.1

[6] Klokman VW, Barten DG, Peters NALR, Versteegen MGJ, Wijnands JJJ, van Osch FHM, Gaakeer MI, Tan ECTH, Boin A. A scoping review of internal hospital crises and disasters in the Netherlands, 2000–2020. PLoS ONE. 2021;16:1–13. 10.1371/journal.pone.0250551. 10.1371/journal.pone.0250551 PMC8075216 33901248

[7] Şenol Balaban M, Doğulu C, Akdede N, Akoğlu H, Karakayalı O, Yılmaz S, Yılmaz S, Ajobiewe T, Güzel S, İkizer G, Akin M, Ünal Y, Karancı AN. Emergency response, and community impact after February 6, 2023 Kahramanmaraş Pazarcık and Elbistan Earthquakes: Reconnaissance findings and observations on affected region in Türkiye. Bull Earthq Eng. 2025;23:1053–81. 10.1007/s10518-024-01867-3

[8] Kapisiz A, Kaya C, Eryılmaz S, Azzam A, Sevimli A, Karabulut R, Turkyilmaz Z, Sonmez K. Observations and experiences of pediatric surgeons working on the field in the first 7 days of the Kahramanmaraş earthquake. Ann Surg Treat Res. 2023;105:114–7. 10.4174/astr.2023.105.2.114. 37564947 10.4174/astr.2023.105.2.114 PMC10409630

[9] İlhan B, Eroǧlu O, Çanak H, Arlkan A, Sakalll M, Tursun S, Deniz T. The Utilization of emergency department and outpatient clinics among evacuated victims after the 2023 Turkey Earthquake, Prehosp. Disaster Med. 2024;39:20–4. 10.1017/S1049023X2300674X 10.1017/S1049023X2300674X 38192268

[10] Köseoğlu Z, Çolak T, Beydilli İ, Altunok G, Şener K, Demir K, Uzan A, Söker S. Data analysis of patients admitted to the emergency medicine clinic of Mersin City Training and Research Hospital after the Kahramanmaraş earthquake. Ulus Travma ve Acil Cerrahi Derg. 2024;30:579–87. 10.14744/tjtes.2024.68523. 10.14744/tjtes.2024.68523 PMC11372496 39092969

[11] Karimiyan A, Khankeh HR, Dalvandi A, Farzin Nia B. The effect of teaching principles of hospital preparedness according to the national program on preparedness of Shahid Motahari Burns Hospital of Tehran in Response to Disasters, Heal. Emergencies Disasters Q. 2017;2:25–32. 10.18869/nrip.hdq.2.1.25

[12] Mulyasari F, Inoue S, Prashar S, Isayama K, Basu M, Srivastava N, Shaw R. Disaster preparedness: Looking through the lens of hospitals in Japan. Int J Disaster Risk Sci. 2013;4:89–100. 10.1007/s13753-013-0010-1.

[13] Altun U, Ceylan H. Sağlıkta tesis yönetiminin afet, acil durum ve risk yönetimi boyutlarıyla incelenmesi: Güvenli hastaneler, Sinop Üniversitesi Boyabat İktisadi ve İdari Bilim. Fakültesi. 2023;3:4–41. https://izlik.org/JA86TA27RH

[14] Munasinghe NL, O’Reilly G, Cameron P. Establishing the domains of a Hospital Disaster Preparedness Evaluation Tool: A systematic review, Prehosp. Disaster Med. 2022;37:674–86. 10.1017/S1049023X22001212 10.1017/S1049023X22001212 PMC9470528 36052843

[15] Palteki T, Aydın E, Saral BZ. Bibliometric analysis of studies conducted in the field of hospital disaster plan between 2002–2021. Resilience. 2023;7:111–22. 10.32569/resilience.1204313

[16] Al-Hajj S, Abou-El-Hassan H, Khalil L, Kaafarani HM, Sayed ME. Hospital disaster and emergency preparedness (HDEP) in Lebanon: A national comprehensive assessment. Int J Disaster Risk Reduct. 2020;51:101889. 10.1016/j.ijdrr.2020.101889.

[17] Samei B, Babaie J, Sadegh Tabrizi J, Sadeghi-Bazargani H, Azami-Aghdash S, Derakhshani N, Rezapour R. Factors affecting the functional preparedness of hospitals in response to disasters: A systematic review. Bull Emerg Trauma. 2023;11:109–18. 10.30476/BEAT.2023.97841.1414 37525651 10.30476/beat.2023.97841.1414 PMC10387338

[18] Naser WN, Saleem HB. Emergency and disaster management training; knowledge and attitude of Yemeni health professionals- a cross-sectional study. BMC Emerg Med. 2018;18:1–12. 10.1186/s12873-018-0174-5. 30081832 10.1186/s12873-018-0174-5 PMC6091203

[19] Usta G, Torpuş K. Evaluation of the disaster preparedness level of a flood-affected hospital: Turkey. Int J Disaster Risk Reduct. 2024;110:104581. 10.1016/j.ijdrr.2024.104581.

[20] De La Garza C, Lot N. The socio-organizational and human dynamics of resilience in a hospital: The case of the COVID-19 crisis. J Contingencies Cris Manag. 2022;30:244–56. 10.1111/1468-5973.12419. 10.1111/1468-5973.12419 PMC9349581 40477987

[21] Moradi SM, Nekoei-Moghadam M, Abbasnejad A, Hasheminejad N. Risk analysis and safety assessment of hospitals against disasters. J Educ Health Promot. 2021;10:412. 10.4103/jehp.jehp_1670_20. 35071618 10.4103/jehp.jehp_1670_20 PMC8719538

[22] The Organisation for Economic Co-operation and, Development OECD, Topics-Health OECD. (n.d.). https://www.oecd.org/en/topics/health.html.

[23] United Nations Office for Disaster Risk Reduction, Sendai Framework for Disaster Risk Reduction 2015–2030. (2015). https://www.unisdr.org/files/43291_sendaiframeworkfordrren.pdf. (accessed April 5, 2019).

[24] Ministry of Health of the Republic of Türkiye, Health Quality Standards, Hospital. Health Services General Directorate Health Quality, Accreditation and Employee Rights Department, Ankara. 2020. https://dosyamerkez.saglik.gov.tr/Eklenti/41258/0/skshastane-seti-s-61--09082021pdf.pdf.

[25] Hospital Disaster and Emergency Plans Implementation Regulation, Hospital Disaster and Emergency Plans (HAP) Implementation Regulation. Date: 18 March 2020 Wednesday, No: 31072. (2020). https://www.resmigazete.gov.tr/eskiler/2020/03/20200318-2.htm.

[26] Fraenkel JR, Wallen NE, Hyun HH. How to design and evaluate research in education. 8th ed. New York: McGraw-Hil; 2012.

[27] Azarmi S, Pishgooie AH, Sharififar S, Khankeh HR, Hejrypour SZ. Challenges of hospital disaster risk management: A systematic review study, Disaster Med. Public Health Prep. 2022;16:2141–8. 10.1017/dmp.2021.203 10.1017/dmp.2021.203 34429178

[28] Zhong S, Clark M, Hou X, Zang Y, Fitzgerald G. Validation of a framework for measuring hospital disaster resilience using factor analysis. 2014:6335–53. 10.3390/ijerph110606335. 10.3390/ijerph110606335 PMC4078582 24945190

[29] Dinçer S, Kumru S. Afet ve acil durumlar için sağlık personelinin hazırlıklı olma durumu, Gümüşhane Üniversitesi Sağlık Bilim. Derg. 2021;10:32–43. 10.37989/gumussagbil.790884

[30] Lestari F, Paramitasari D, Kadir A, Firdausi NA, Hamid AY, El-matury HJ, Wijaya O, Ismiyati A. The application of hospital safety index for analyzing primary healthcare center (PHC) disaster and emergency preparedness. Sustain. 2022;14:1–19. 10.3390/su14031488

[31] Achour N, Pascale F, Price ADF, Polverino F, Aciksari K, Miyajima M, Özüçelik DN, Achour N, Pascale F, Price ADF, Polverino F. Learning lessons from the 2011 Van Earthquake to enhance healthcare surge capacity in Turkey. Environ Hazards. 2016;0:1–21. 10.1080/17477891.2016.1139539.

[32] De Almeida MM. Recovering, not recovered Hospital disaster resilience : A case-study from the 2015 earthquake in Nepal, Glob. Health Action. 2022;15. 10.1080/16549716.2021.2013597 10.1080/16549716.2021.2013597 PMC8843347 35138232

[33] Skryabina E, Betts N, Reedy G, Riley P, Amlôt R. UK healthcare staff experiences and perceptions of a mass casualty terrorist incident response: A mixed-methods study. Emerg Med J. 2021;38:756–64. 10.1136/emermed-2019-208966. 33177061 10.1136/emermed-2019-208966 PMC8461407

[34] O’brien RM. Regarding rules of thumb for variance inflation factors. Qual Quant. 2007;41:673–90. 10.1007/s11135-006-9018-6

[35] Hosmer DW, Lemeshow S, May S. Applied Survival Analysis: Regression Modeling of Time to Event Data. Second: Wiley, Canada,; 2008.

[36] Sarık ME, Cengiz S. Hastane afet ve acil durum planı eğitim, hazırlık düzeyi ve çalışanların bilgi seviyelerinin tespit edilmesi: Antalya ili örneği, Gümüşhane Üniversitesi Sağlık Bilim. Derg. 2022;11:122–32. 10.37989/gumussagbil.1003368

[37] Avcı S, Kaplan B, Ortabağ T, Arslan S. Üniversite hastanesinde çalışan hemşirelerin afet konusundaki bilgi ve bilinç düzeyleri, Afet ve Risk Derg. 2022;5:94–108. 10.35341/afet.1034678

[38] Şen G, Ersoy G. Hastane afet ekibinin afete hazırlık konusundaki bilgi düzeylerinin değerlendirilmesi, Gümüşhane Üniversitesi Sağlık Bilim. Derg. 2017;6:122–30. https://dergipark.org.tr/en/pub/gumussagbil/issue/32215/366190

[39] Paganini M, Borrelli F, Cattani J, Ragazzoni L, Djalali A, Carenzo L, Della Corte F, Burkle FMJ, Ingrassia PL. Assessment of disaster preparedness among emergency departments in Italian hospitals: A cautious warning for disaster risk reduction and management capacity. Scand J Trauma Resusc Emerg Med. 2016;24:1–8. 10.1186/s13049-016-0292-6. 27526719 10.1186/s13049-016-0292-6 PMC4986169

[40] Sa’d RI, Malak MZ. The effect of disaster management training program on emergency nurses’ knowledge, skills, and personal preparedness in Palestine. Int Emerg Nurs. 2025;80. 10.1016/j.ienj.2025.101601. 10.1016/j.ienj.2025.101601 40088614

[41] Ali HM, Ranse J, Roiko A, Desha C. Health care workers’ perceptions of hospital disaster planning and preparedness for building resilient healthcare systems. Disaster Med. Public Health Prep. 2025:19. 10.1017/dmp.2025.3. 10.1017/dmp.2025.3 40160147

[42] Calvi DPPM, Fischer RNEC. Seismic performance of non – structural elements during the 2016 Central Italy earthquake. Bull Earthq Eng. 2019;17:5655–77. 10.1007/s10518-018-0361-5.

[43] Qu Z, Wang F, Chen X, Wang X, Zhou Z. Rapid report of seismic damage to hospitals in the 2023 Turkey earthquake sequences. Earthq Res Adv. 2023;3:100234. 10.1016/j.eqrea.2023.100234.

[44] Pamuk Cebeci S, Arberk OK.Healthcare professionals hospital disaster and emergency situation plan knowledge level . J Acad Soc Sci. 2021;9:103–12. 10.29228/ASOS.52169

[45] Gerdan S, Yazıcı A, Yılmaz S, Çağlayan Ç. Sustainability of health services in a üniversity hospital during disaster: The Kahramanmaraş Earthquakes experience. Bezmialem Sci. 2025;13:334–41. 10.14235/bas.galenos.2025.25993

[46] AslanR. Dispatched into disaster: aA qualitative study on medical rescue teams’ personnel’s preparation, mobilization, and field living conditions after the Kahramanmaraş earthquakes. 2025;25:1–11. 10.1186/s12873-025-01416-4 10.1186/s12873-025-01416-4 PMC12751924 41462101

[47] Seyghalani Talab F, Ahadinezhad B, Khosravizadeh O, Amerzadeh M. A model of the organizational resilience of hospitals in emergencies and disasters. BMC Emerg Med. 2024;24:1–13. 10.1186/s12873-024-01026-6. 38914937 10.1186/s12873-024-01026-6 PMC11197230

[48] Altuntaş S, Çiçek Korkmaz A, Efe N, Demirtaş H, Kuleyin B. Problems experienced by nurses working in the region affected by Kahramanmaraş earthquakes in Turkey and their recommendations for risk reduction: A descriptive and cross-sectional study. Int J Disaster Risk Reduct. 2023;99:104115. 10.1016/j.ijdrr.2023.104115.

[49] Taşkın Ö, Dişel NR. From tragedy to resilience in a university hospital: Characteristics of patients in the aftermath of the 2023 Turkey Earthquake. Disaster Med Public Health Prep. 2024;18. 10.1017/dmp.2024.52 10.1017/dmp.2024.52 38602095

[50] Mishra KG, Patnaik N, Pradhan NR, Mohapatra A, Saleem SM. Comparative descriptive analysis of hospital disaster preparedness using WHO safety index: A multi-center study from Eastern India. BMC Emerg Med. 2025;25. 10.1186/s12873-025-01248-2 10.1186/s12873-025-01248-2 PMC12505759 41062974

[51] Yılmaz S, Karakayali O, Yilmaz S, Çetin M, Eroglu SE, Dikme O, Özhasenekler A, Orak M, Yavaşi Ö, Karbek Akarca F, Günalp Eneyli M, Erbil B, Akoğlu H. Emergency Medicine Association of Turkey Disaster Committee summary of field observations of February 6th Kahramanmaraş Earthquakes, Prehosp. Disaster Med. 2023;38:415–8. 10.1017/S1049023X23000523 10.1017/S1049023X23000523 37198906

