# Supplementary material for: Factors affecting hospital personnel’s disaster management performance
Source: BMC Emerg Med. 2026 Apr 24;26:175. doi: 10.1186/s12873-026-01597-6 (PMC13277243; doi:10.1186/s12873-026-01597-6)
Supplement: Supplementary file 1 — Supplementary Material 1 [file 12873_2026_1597_MOESM1_ESM.docx]

**SURVEY FORM**

This survey form has been prepared for the study titled "Factors Affecting Hospital Disaster Management Performance". It is not necessary to disclose names for this research. The data obtained from this survey will be used solely for scientific purposes.

Master's Student: Burak KAYA

Academic Advisor: Dr. Sefa MIZRAK

**Part 1**

**1. What is your gender?**

(1) Male

(2) Female

**2. How old are you?**

(1) 20-25

(2) 26-30

(3) 31-35

(4) 36 and over

**3. What is your marital status?**

(1) Married

(2) Single

**4. What is your educational level?**

(1) Secondary education or below

(2) High school

(3) Preundergraduate

(4) Undergraduate

(5) Master's degree

(6) Doctorate

**Part 2**

**1. How many years have you been working in your profession?**

(1) 1-3 years

(2) 4-10 years

(3) 11 years or above

**2. What is your role in this institution?**

(1) Medical doctor

(2) Nurse

(3) Cleaning staff

(4) Secretary

(5) Technician

(6) Other health graduates

(7) Officer

(8) Security guard

**3. On which floor of the hospital do you work?**

(1) Basement floor

(2) Ground floor

(3) 1st floor

(4) 2nd floor

(5) 3rd floor

(6) 4th floor

(7) 5th floor

(8) 6th floor

**4. What are your usual working hours at this hospital?**

(1) 8 hours

(2) 12 hours

(3) 24 hours

**5. Have you received any disaster education?**

(1) No

(2) I received insufficient disaster education

(3) I received intermediate-level disaster education

(4) I received sufficient disaster education

**6. How much do you know about your hospital's disaster plans?**

(1) I have no knowledge

(2) I have intermediate knowledge

(3) I have a lot of knowledge

**7. Do you have a current role in the hospital's disaster and emergency plans?**

(1) I have no duty

(2) Response team

(3) Rescue team

(4) Incident management team

(5) CBRN team

(6) I do not know if I have a duty or not

**Part 3**

**1. Items within the hospital are arranged appropriately against disaster risks (for example, cabinets are fixed, devices are fixed).**

(1) I strongly disagree

(2) I disagree

(3) Undecided

(4) I agree

(5) I strongly agree

**2. I know what to do in case of a disaster in the hospital.**

(1) I strongly disagree

(2) I disagree

(3) Undecided

(4) I agree

(5) I strongly agree

**3. In case of a disaster, we can work in coordination with the personnel in my unit.**

(1) I strongly disagree

(2) I disagree

(3) Undecided

(4) I agree

(5) I strongly agree

**4. In case of disaster, our hospital staff can work in coordination with other institution personnel (Disaster and Emergency Management Directorate, Gendarmerie, Police, Fire Brigade, etc.).**

(1) I strongly disagree

(2) I disagree

(3) Undecided

(4) I agree

(5) I strongly agree

**5. I think that in case of a disaster, the materials in my unit will be sufficient for at least three days.**

(1) I strongly disagree

(2) I disagree

(3) Undecided

(4) I agree

(5) I strongly agree

**6. In case of a disaster, your hospital personnel's disaster management performance will be successful (Imagine a major disaster in Giresun)**

(1) I strongly disagree

(2) I disagree

(3) Undecided

(4) I agree

(5) I strongly agree
